# Ultrasonographic aspects of the Achilles tendon after tenotomy for the treatment of congenital clubfoot by the Ponseti technique

**DOI:** 10.1590/0100-3984.2018.0086

**Published:** 2020

**Authors:** Marcelo de Toledo Piza Watzl, Alair Augusto Sarmet Moreira Damas dos Santos, Armando Leão Ferreira Neto, Danilo Alves de Araujo

**Affiliations:** 1 Universidade Federal Fluminense (UFF), Niterói, RJ, Brazil.; 2 Universidade do Estado do Rio de Janeiro (UERJ), Rio de Janeiro, RJ, Brazil.

## INTRODUCTION

The Ponseti technique consists in percutaneous tenotomy of the Achilles tendon for correction of congenital talipes equinovarus (clubfoot). The healing of the Achilles tendon can be evaluated by ultrasound.

**Figure 1:**
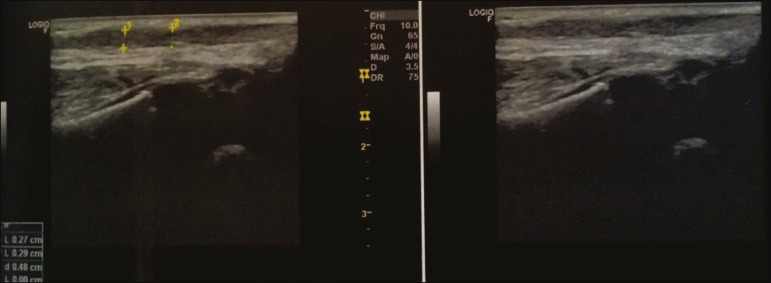
Longitudinal images of an Achilles tendon, showing scar formation consistent with the final phase of healing.

**Figure 2:**
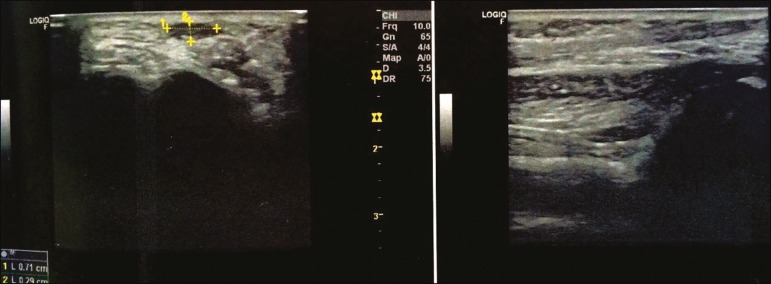
Transverse and longitudinal views of an Achilles tendon in the final phase of healing, showing a hypoechoic area, measuring 0.71 × 0.29 cm.

Ultrasound is a dynamic, noninvasive tool for the assessment of the severity of clubfoot and the healing of the Achilles tendon, allowing its regeneration to be monitored, as well as allowing the thickness of the tendon and the length of the reparative tissue to be quantified^(1-6)^ . The ultrasound examination of individuals with clubfoot involves the use of a high-resolution multifrequency linear transducer to obtain longitudinal and transverse images of the Achilles tendon (Figures 1 and 2, respectively), evaluating the regeneration of the tendon in terms of its thickness and echogenicity, the peritendinous structures, the gap between the stumps, and adherence of the skin to the tendon.

After tenotomy of the Achilles tendon, there is a separation, and the space between the tendons caused by the retraction of the proximal stump is filled in by a hematoma, characterized by echogenic debris, which evolves and becomes fibrous tissue that is easily seen on ultrasound. The phases of the tendon healing process can be divided into the inflammatory phase, in which the hematoma forms and subsequently organizes; the proliferative phase, in which there is growth and maturation of connective tissue fibers in the gap; and the remodeling phase, in which with the tendinous structure organizes into tissue similar to the original^(1,4)^ .

## ULTRASONOGRAPHIC ASPECTS

Here, we describe each stage of the healing of an Achilles tendon treated with the Ponseti technique for the correction of congenital clubfoot, together with the related ultrasonographic aspects:

- Prior to tenotomy: normal tissue with well-defined margins and a fibrillar texture^(3)^ ;
- 1 to 2 weeks after tenotomy: mixed echogenicity with a heterogeneous appearance, corresponding to a hematoma^(3,5,6)^ ;
- 2 to 3 weeks after tenotomy: filling of the gap with irregular hypoechoic tissue;
- 3 to 6 weeks after tenotomy: fibers randomly arranged within the gap and continuity between the tendinous segments on dynamic studies;
- 6 to 12 weeks: fibers with a linear appearance, although fewer in number and less hyperechoic than those in a normal tendon^(3,5,6)^ ;
- After week 12: homogeneous fibrillar appearance similar to that of a normal tendon, in terms of echogenicity and dimensions^(3,6)^ .

## CONCLUSION

Ultrasound is accepted as a reliable method for the routine assessment of tendons, which allows a dynamic assessment and is extremely useful in evaluating regeneration of the Achilles tendon after Ponseti tenotomy. The initial post-tenotomy phase reveals mixed echogenicity and a heterogeneous appearance of continuity between the two ends, which is thereafter replaced by irregular hypoechoic tissue and subsequently by fibers arranged randomly in the gap. Six months after the procedure, the fibers are linear in appearance, fewer in number, and less echogenic than those of the normal tendon, gradually becoming homogeneous and normal in appearance and size.

Ultrasound evaluation makes it possible to track the healing and repair of the Achilles tendon after Ponseti tenotomy in patients with congenital clubfoot. It is therefore important for the radiologist to know more about this application of ultrasound.
